# The Hospital and Nursing Exhibition

**Published:** 1923-03

**Authors:** 


### THE HOSPITAL EXHIBITION.

ARRANGEMENTS for the Hospital Nursing and Midwifery Exhibition are now making rapid progress, and there is every reason to anticipate a success even greater than on previous occasions. For the first time the exhibition becomes the " Hospital, Nursing and Midwifery Exhibition," and the inclusion of the hospital element marks a definite advance in scope and interest. Hitherto this familiar fixture of the early part of the season has been held at the Horticultural Hall, but it has now outgrown the accommodation which formerly sufficed, and this year it will occupy the whole of two spacious floors of the Central Hall, Westminster, from Easter Tuesday, April 3, to the following Saturday. The Reception Committee will include the Hon. Sir Arthur Stanley, G.B.E., treasurer of St. Thomas's Hospital; Sir Napier Burnett, K.B.E., Director of Hospital Service to the British Red Cross Society; Lady Acland, and other persons of distinction interested in public health and hospital subjects.

### The Cinema in the Hospitals.

The ground floor of the Central Hall, which will hold more than 2,700 people, has been secured for a novel purpose. Mr. Godfrey H. Hamilton, Secretary of the National Hospital for the Paralysed and Epileptic, will there lecture on the opening day from 3.30 to 4.30 on Hospital Administration, with cinematograph illustrations. These films will be of remarkable and comprehensive interest. They include pictures of the wards of many of the great hospitals, and of the Seamen's Hospital, at Greenwich, taken for the purpose by Mr. Joseph Best, B.Sc. There will also be films showing the latest X-ray devices, clinics, laboratories, operating theatres and dispensaries, nursing work, bandaging and midwifery subjects, and it is hoped that similar illustrations from Continental and American hospitals will be obtainable in time, by way of contrast and comparison with our own methods. The educative values of films of this character need not be insisted upon; it is obvious that they will add greatly to the interest and the professional value of Mr. Hamilton's lecture.

### Opening Day Invitations.

The large number of invitations which will be issued for the opening day to the medical and administrative staffs of hospitals readily accessible to London—a phrase which naturally includes many of the great towns—will give free admission from 2 p.m. to 6 p.m., and tea will be provided for the whole of this comprehensive party of guests. The exhibition thus promises not only to be by far the most generally interesting of the series which began in 1908, but to be of greatly enhanced value to those who, whether as medical men, nurses, or in other ways, are professionally concerned with the perfecting of hospital and nursing systems and the advancement of the public health. Tickets may be obtained upon application, enclosing a stamped and addressed envelope, to the Secretary of the Exhibition at 22 and 24, Great Portland Street, W.1.

